# LRP5 enhances glioma cell proliferation by modulating the MAPK/p53/cdc2 pathway

**DOI:** 10.7150/ijms.99920

**Published:** 2025-02-03

**Authors:** Ying-Yi Feng, Xin Jin, Min-Xuan Pan, Jia-Min Liao, Xian-Zhang Huang, Chun-Min Kang

**Affiliations:** 1 The Second Clinical College of Guangzhou University of Chinese Medicine, Guangzhou, Guangdong, 510120, China.; 2 Department of Laboratory Medicine, Guangdong Provincial Hospital of Chinese Medicine, Guangzhou, Guangdong, 510120, China.; 3 State Key Laboratory of Dampness Syndrome of Chinese Medicine, Guangzhou, Guangdong, 510120, China.; 4 Guangdong Provincial Key Laboratory of Clinical Research on Traditional Chinese Medicine Syndrome, Guangzhou, Guangdong, 510120, China.; 5 Department of Neurosurgery, Guangdong Sanjiu Brain Hospital, Guangzhou, Guangdong, 510120, China.

**Keywords:** glioma, LRP5, G2/M phase, cell cycle progression, Cyclin B-cdc2 complex

## Abstract

**Background:** Glioma is a malignant neoplasm with generally poor prognosis and the treatment options and effective drugs are very limited. LRP5, a member of the low-density lipoprotein receptor (LDLR) gene family, has been reported to regulate the progression of various cancers such as gastric and colorectal cancer. However, the function of LRP5 in glioma has not been elucidated. The objective of this study is to explore the influence of LRP5 in glioma cell proliferation and its potential molecular mechanisms.

**Methods:** LRP5 expression in glioma was assessed through bioinformatics analysis, and validation was conducted using clinical glioma tissues. Glioma cell lines with reduced LRP5 expression were established through RNA interference. A series of experiments such as cell proliferation assay, flow cytometry analysis, and Western blotting were used to determine the role of LRP5 in glioma cell proliferation, cell cycle progression, and the underlying mechanisms.

**Results:** LRP5 was found to be upregulated in glioma tissues and exhibited significant variations across various subtypes of glioblastoma (GBM). When differentiating between normal individuals and glioma patients, the area under the receiver operating characteristic curve (ROC) for LRP5 was determined to be 0.981. Downregulating the expression of LRP5 in glioma cells can weaken their proliferative ability and reduce the number of cell colonies. There were more cells arrested in the G2/M phase of the cell cycle. The protein levels of phospho-p53 (p-p53), p21^Cip1^, and phospho-cdc2 (p-cdc2) were elevated. Moreover, LRP5 down-regulation suppressed the phosphorylation of the mitogen-activated protein kinase (MAPK) family members, JNK and p38 MAPK. Consistent results with those mentioned above can be achieved by using an LRP5 antagonist named DKK-1.

**Conclusion:** This research has identified that LRP5 may promote glioma proliferation by influencing the G2/M transition and the activation of the MAPK/p53/cdc2 pathways, suggesting its value as a potential molecular target for glioma diagnosis and treatment.

## Introduction

Glioma is a common primary malignant intracranial tumor with a high recurrence rate and a poor prognosis.^1^ In the WHO classification, glioma can be divided into four grades. The most severe form, Grade IV, is known as glioblastoma (GBM). The median survival period of patients with glioblastoma is about 15 months, and the 5-year survival rate is 6.8%.^2^ Isocitrate dehydrogenase (IDH) mutations are mainly found in low-grade glioma (LGG), which include grade I and grade II glioma. Glioma with codeletion of chromosome arms 1p and 19q (1p/19q co-deleted) are mainly detected in oligodendrogliomas.^3^ Treatments for glioma mainly include surgery, chemotherapy, and radiotherapy. The combination of temozolomide (TMZ) and radiotherapy can improve survival rates.^4^ However, the effectiveness of the above methods is not ideal due to various reasons, such as the high invasiveness of tumor cells, significant heterogeneity between tumors and within tumors, blood-brain barrier, immune-suppressive microenvironment, drug resistance mechanisms, etc.^5^, ^6^ Furthermore, the fundamental mechanisms underlying the development of glioma are not fully understood and require further investigation.

In the classic Wnt signaling pathway, the Frizzled (FZD) family receptors and LRP5/LRP6 co-receptors transduce Wnt signals to activate the β-catenin signaling cascade. It is essential for cell proliferation and strongly related to the occurrence and progression of cancer.^7^ A study has shown that inhibiting the Wnt pathway can suppress the growth of glioma cells.^8^ LRP5 is one of the low-density lipoprotein receptor (LDLR) family members, which acts as a coreceptor with LRP6 and plays an important role in the Wnt signaling pathway.^9^ In our initial investigation, we utilized gene chip technology to evaluate differential gene expression. The analysis revealed a more than two-fold increase in the expression of the LRP5 gene in glioma tissue compared to adjacent paracancerous tissue. Moreover, there have been literature reports on the relationship between LRP5 and various cancer diseases.^10^-^12^ The potential significance of LRP5 in glioma remains relatively underexplored, with limited existing research on the relationship between LRP5 and glioma, particularly regarding its influence on glioma cell proliferation. LDLR has been identified as a promising target for nanomedicine development in the context of glioma.^13^ Consequently, our study aims to examine the involvement of LRP5 and its associated regulatory pathways in glioma cells, thereby establishing a foundation for further investigation in this area.

In this study, we found that LRP5 shows increased expression levels within glioma tissues, and a decrease in LRP5 expression resulted in a slowdown in cell proliferation. Through cell cycle analysis and western blotting, we found that LRP5 expression promotes the progression of glioma cells by influencing the transition from the G2 phase to the M phase. The effect of LRP5 on glioma cell proliferation may be related to the activation of the JNK and p38 pathways. These findings illustrate the relationship between LRP5 and glioma and elucidate the potential regulatory mechanisms involved.

## Materials & Methods

### Bioinformatics analysis

We downloaded all the RNA-Seq gene expression data and clinical data from the TCGA and GTEx databases. The expression of LRP5 in pan-cancer was analyzed using the data from the TCGA database, while its expression in GBM and LGG was analyzed through the GEPIA2 online tool. In the GBM group, 518 tumor tissues and 207 normal tissues were collected, while in the LGG group, there were 163 tumor tissues and 207 normal tissues. LRP5 expression was investigated among different WHO subtypes, IDH mutation and wild type, and 1p/19q co-deletion and wild type were investigated using 669 RNA-Seq of GBM from the TCGA database. Finally, the ability of LRP5 to distinguish between GBM tissues and normal tissues was analyzed using the receiver operating characteristic (ROC) curve. The data of the GBM group were selected from the TCGA database and that of the normal group were extracted from the GTEx database.

### Cell culture, transfection, and treatment

Human glioblastoma cell lines A172 and SHG-44 were obtained from the American Type Culture Collection (ATCC). The cells were cultured at 37°C with 5% CO2 using Dulbecco's Modified Eagle's Medium (DMEM) (Gibco, USA) containing 10% Fetal Bovine Serum (FBS) (Gibco, USA). A172 and SHG-44 cells with LRP5 or negative control (NC) knockdown were then constructed using lentiviral transfection. The sh-NC and sh-LRP5 lentiviruses were purchased from Guangzhou RiboBio Co., Ltd. We plated cells at the period of exponential growth phase in plates and cultured them for 24h. Then they were transfected with sh-NC and sh-LRP5 lentiviruses and subsequently screened with puromycin to obtain stably transfected cells.^14^ DKK-1 was obtained from MedChemExpress (MCE, HY-P72968). A172 and SHG-44 cells were treated with or without 0.5μg/mL DKK-1 for 48h.

### Immunohistochemistry (IHC)

The paraffin sections of brain tissue are collected from 6 patients with glioma. Sections were deparaffinized with xylene followed by rehydration through graded alcohol. Then they were treated with citrate buffer (pH 6.0) for antigen retrieval and incubated with 3% H_2_O_2_ to inhibit endogenous peroxidase. Sections were incubated in 4% goat serum for blocking and then wiped off. LRP5 antibody (1:200, Invitrogen) or Ki67 antibody (1:200, Abcam) was applied to the sections and incubated overnight at 4°C. After that, sections were incubated in secondary antibody (DAB Detection Kit, GK500710, Gen Tech) for 30 minutes at room temperature and dyed with DAB for 1.5 minutes. Tissues were counterstained with hematoxylin for 3 minutes and differentiated in a solution of 1% hydrochloric acid in absolute ethanol for 2 seconds. Finally, tissues were dehydrated, mounted with neutral balsam, and then covered with coverslips. The negative control was exposed to 4% goat serum. Stained sections were observed under a microscope and taken pictures. Image J was used to calculate the average percentage of positive cells. 15 This study was approved by the Ethics Committee of Guangdong Sanjiu Brain Hospital (Approval number: 2021-01-084; Approval data: 2021-11-24). Written informed consent was obtained from all participants.

### Western blotting

The tissues and cultured cells were collected and lysed in lysis buffer. The concentration of protein was determined by the BCA method. Total protein was separated by 10% SDS-PAGE and transferred onto a polyvinylidene difluoride (PVDF) membrane. The membranes were blocked with 2% BSA blocking buffer for 2 hours at room temperature and incubated in corresponding primary antibodies overnight at 4°C. The membranes were incubated with secondary antibody at room temperature for 1 hour. After development, the results were detected by Tanon5200 Automatic chemiluminescence image analysis system, and the images were analyzed with GAPDH as the reference through Image J. Antibodies applied in WB assay were listed as follows: LRP5 (CST; 5731S); p53 (CST; 48818S); phospho-p53 (CST; 9286S); p21^Cip1^ (CST; 37543S); cdc2 (CST; 77055S); phospho-cdc2 (CST; 4539S); JNK (Proteintech; 24164-1-AP); phospho-JNK (Proteintech; 80024-1-RR); p38 (CST; 8690T); phospho-p38 (CST; 4511T); ERK1/2 (CST; 4695T); phospho- ERK1/2 (CST; 4376T); GAPDH (CST, 5174S).

### CCK8 assay

CCK8 assay was performed using Cell Counting Kit-8 (Dojindo Laboratory, CK04). A172 or SHG-44 cells were seeded in 96-well plates and transfected with sh-RNA. After culturing the cells for an appropriate time (48h, 72h, and 96h), CCK-8 solution was added (10μL/well) and cultured for 2 hours at 37°C. The optical density (OD) value at 450nm was measured by a microplate reader.

### EdU assay

The EdU assay was performed using an EdU Kit (Beyotime, C0071S) according to the manufacturer's instructions. Cells were cultured with a 10μM Edu solution for 2 hours, fixed with 4% paraformaldehyde for 15 minutes, and permeabilized with 0.3% Triton X-100 for 10 minutes. The click additive solution was then added, and the cells were cultured for 1 hour in the dark at room temperature. The cell nuclei were stained with Hoechst. After staining, cells were photographed with a fluorescence microscope with the same parameters.^16^

### Cell clone formation

Stably transfected cells were seeded in 6-well plates and cultured for 15 days. Then cells were washed with PBS and stained with crystal violet. The amount of cell colonies was counted under a microscope. The difference in the number of cell colonies between the two groups was calculated with unpaired t test. 17

### Cell cycle analysis

Cells were harvested and resuspended for preparation of single-cell suspensions (1×10^6^/mL) using PBS. 1mL of single-cell suspension was taken and centrifuged, and the supernatant was discarded. Add 70% precooled ethanol to fix the cells overnight at 4°C. Before staining, the fixed cells were washed with PBS, and incubated with 500 μL PI/ RNase staining solution which was pre-prepared (KeyGEN BioTECH, KGA9101-50) at room temperature in the dark for 30 minutes. Flow cytometry was used to detect cell cycle progression, and the results were analyzed with FlowJo 10.8.1.

### Statistical analysis

All experiments were repeated three times, and Student's t test was used to compare quantitative data between groups if they were consistent with normal distribution, or nonparametric tests were used if they were not consistent. *P*
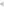
0.05 was considered statistically significant. R Studio was utilized for bioinformatics analysis. The proportion of positive cells of glioma sections with immunohistochemical staining was analyzed by ImageJ. Other statistical analyses were conducted using GraphPad Prism 9.0 and SPSS 26.

## Results

### LRP5 mRNA overexpressed in glioma tissues in bioinformatics analysis

**Figure 1:**
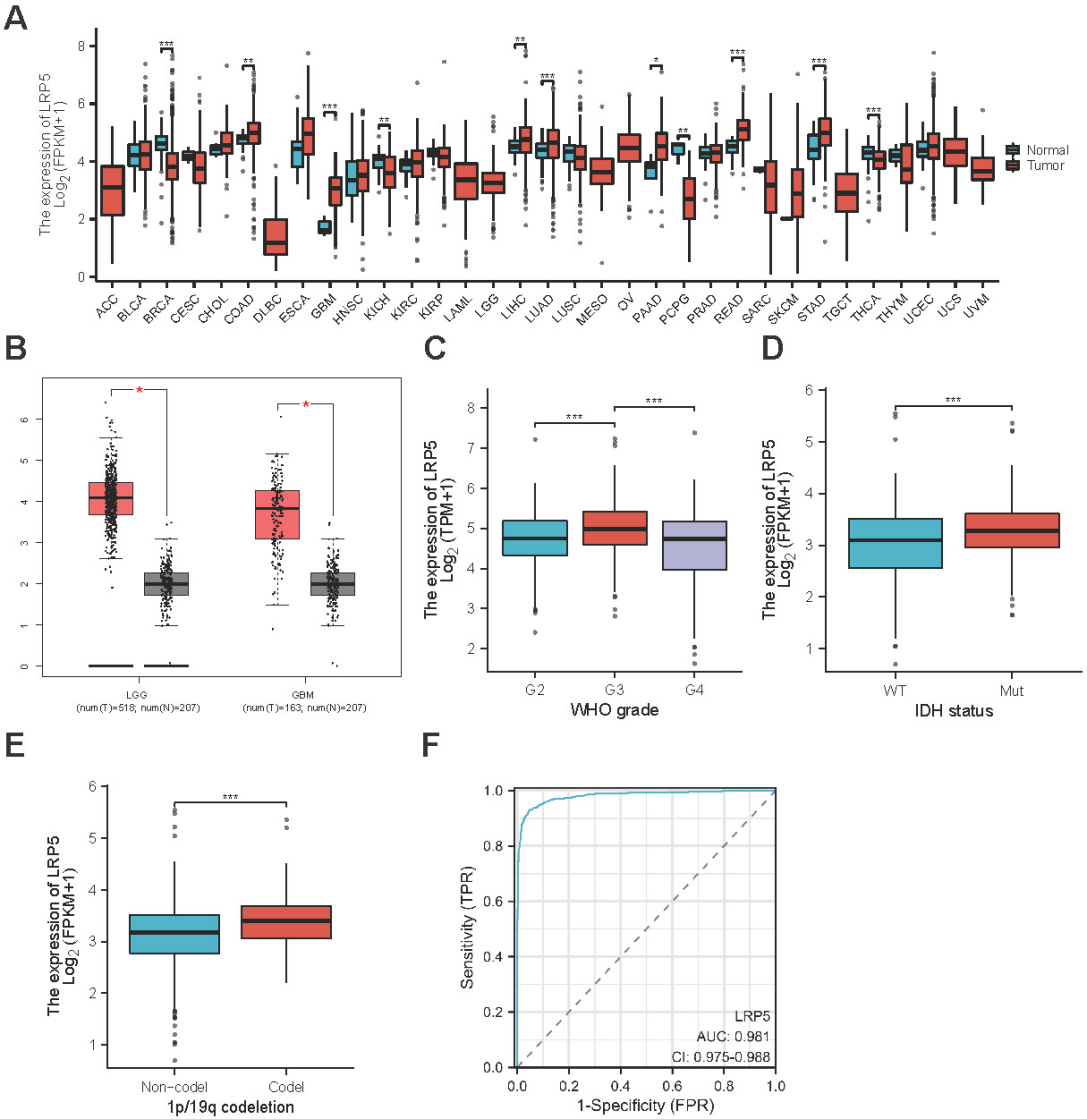
**LRP5 is upregulated in glioma tissues.** (A). Pan-cancer analysis of LRP5 expression. GBM, *** *P* < 0.001. (B). Difference in LRP5 expression in LGG (n=518) and GBM (n=163), respectively, and normal tissues (LGG, n=207; GBM, n=207). GBM, * *P* < 0.05, LGG, * *P* < 0.05. (C). LRP5 expression among different WHO grades of glioma. *** *P* < 0.001. (D). Difference of LRP5 expression between IDH-mutant and wild-type glioma. *** *P* < 0.001. (E). Difference of LRP5 expression between 1p/19q codeleted and non-codeleted glioma. *** *P* < 0.001. (F). ROC curve of LRP5 mRNA levels from GBM tissues and normal controls. AUC = 0.981, 95%CI = 0.975~0.988.

We performed pan-cancer analysis using the clinical data from TCGA databases and found LRP5 mRNA is overexpressed in GBM (Figure 1A). LRP5 expression was elevated in GBM or LGG tumor tissues compared to normal tissues (Figure 1B). Furthermore, LRP5 expression varies significantly among different WHO grades, with higher levels observed in IDH-mutant and 1p/19q co-deleted types compared to their respective control groups (Figure 1C-E). In order to investigate whether LRP5 can serve as a potential biomarker for gliomas, we utilized ROC curves to analyze the capability of LRP5 to differentiate between glioma and normal tissues. The area under the ROC curve (AUC) is 0.981 (95% CI=0.975%~0.988), indicating that LRP5 has good predictive ability and significant diagnostic value in this context (Figure 1F).

### Validation of the protein expression level of LRP5 in clinical glioma tissue

**Figure 2:**
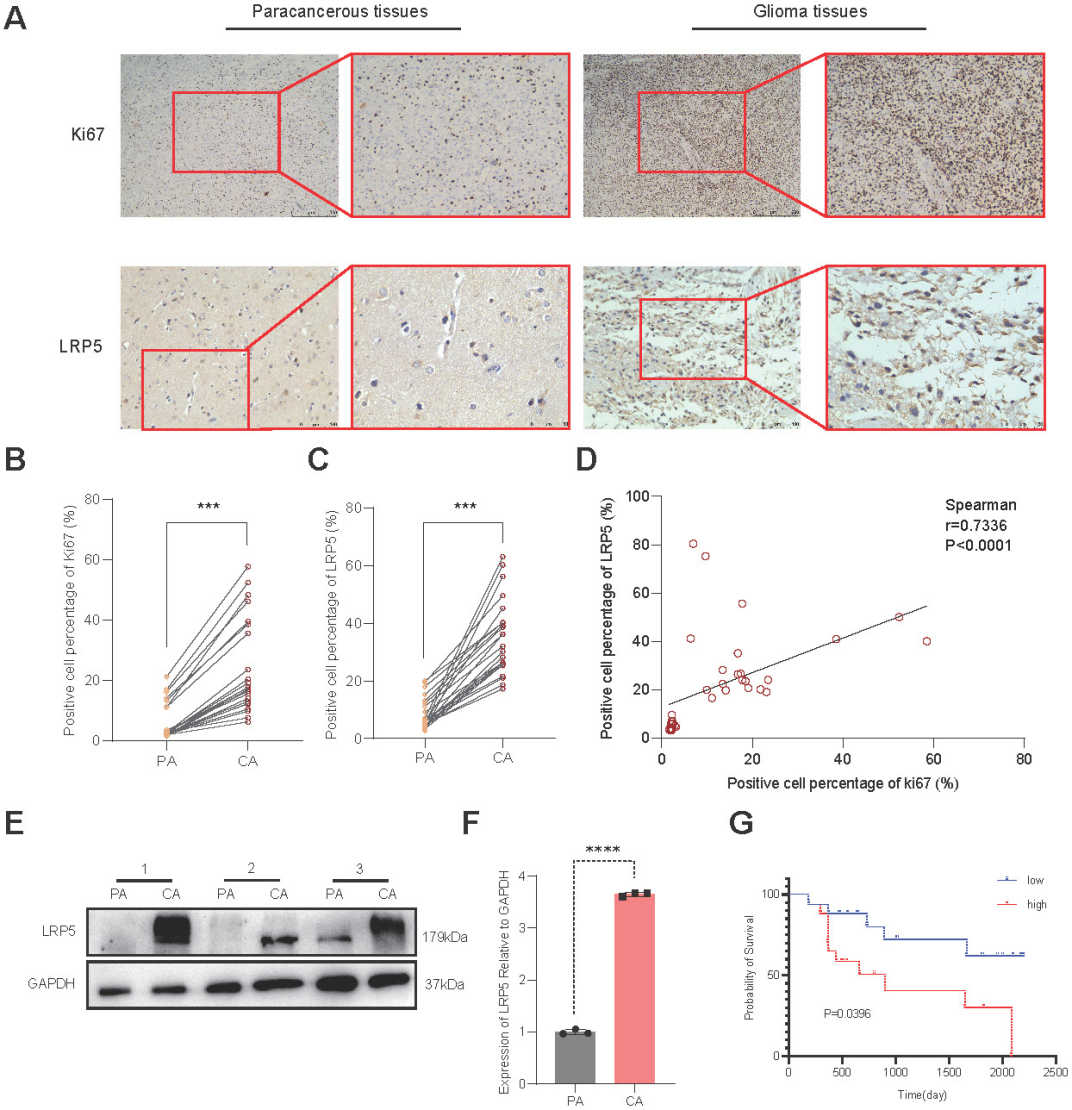
** LRP5 expression in clinical sections and its association with recurrence survival.** (A-C) Representative pictures and the statistical result of glioma and paracancerous tissues after IHC staining of Ki67 and LRP5 (n=6). (Ki67, scale bar=500μm; LRP5, scale bar=100μm) *** *P* < 0.001. (D). The result of correlation analysis between LRP5 and Ki67. r = 0.7336, *P* < 0.0001. (E, F) The relative protein level of LRP5 difference between glioma and paracancerous tissues detected by Western blotting (n=3). **** *P* < 0.0001. (G). Survival analysis result of LRP5 expression with glioma recurrence time. Low: low expression (n=17), High: high expression (n=18). All data were presented as the means ± SD. PA: paracancerous tissues, CA: cancer.

To validate the expression of LRP5 in glioma tissues, we conducted IHC on brain tissue samples from clinical glioma patients. Compared to the paracancerous tissues, the glioma tissues exhibit significantly higher expression of Ki67 and LRP5 (Figure 2A-C). Spearman correlation analysis showed a positive correlation between LRP5 and Ki67 (Spearman correlation coefficient *r* = 0.7336) (Figure 2D), indicating that increased LRP5 expression is highly correlated with abnormal proliferation of glioma cells. This aligns with the outcomes of the Western blotting (Figure 2E-F), providing additional evidence that LRP5 may indeed play a crucial role in the progression of glioma. To assess the utility of LRP5 expression as a predictor of recurrence, 36 clinical sections were used for IHC with LRP5 antibody and then performed survival analysis (the positive event is glioma recurrence) with the results of IHC. The result shows that the overexpression of LRP5 is associated with shorter recurrence-free survival (*P* = 0.0396) (Figure 2G).

### Downregulation of LRP5 inhibited the proliferation of glioma cells

**Figure 3:**
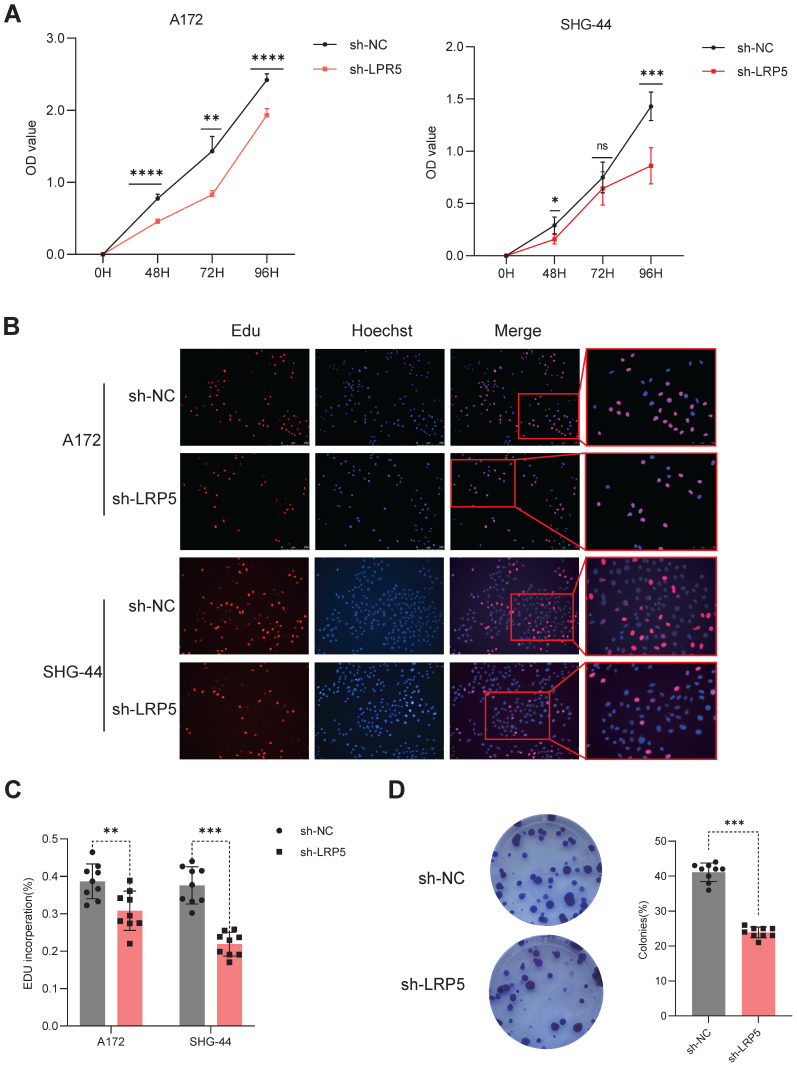
**Downregulated expression of LRP5 inhibited the proliferation of glioma cells.** (A). The result of the CCK8 assay showed that LRP5 silencing significantly reduced the cell viability of A172 and SHG-44 cells. ns *P* = 0.8745, * *P* < 0.05, ** *P* < 0.01, ****P
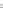
0.0001. (B, C). Representative images and the statistical result of Edu assay in A172 and SHG-44 cells (n=3). The nucleus was stained with Hoechst (blue), and the proliferating cells were stained with Edu (red) (scale bar=250μm). **P
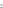
0.01, ***P
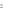
0.001. (D). Representative images and statistical results of cell clone formation assay which reflected glioma cell proliferation. A172 cells, ***P
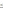
0.001. All data were presented as the means ± SD.

To assess the effect of LRP5 on cell proliferation ability, A172 and SHG-44 cells were transfected with sh-LRP5 or control sh-RNA and then employed to perform CCK8 assay, Edu assay, and cell clone formation assay. After transfection at 48, 72, and 96 hours, the cell proliferation ability of the sh-LRP5 group exhibited a significant depression (Figure 3A). The percentage of Edu-positive cells showed similar changes (Figure 3B-C). The number of colonies in sh-LRP5 A172 cells was markedly reduced compared to the negative control group. (Figure 3D). The above results prove that decreased expression of LRP5 could suppress the proliferation of glioma cells. This finding suggests that the proliferation of glioma cells may be influenced by the overexpression of LRP5. Therefore, LRP5 plays a certain role in the progression of glioma, and the upregulation of LRP5 may promote the growth of tumor cells.

### Silencing of LRP5 induced cell cycle arrest at the G2/M phase and inhibited the activation of JNK and p38 pathways

**Figure 4:**
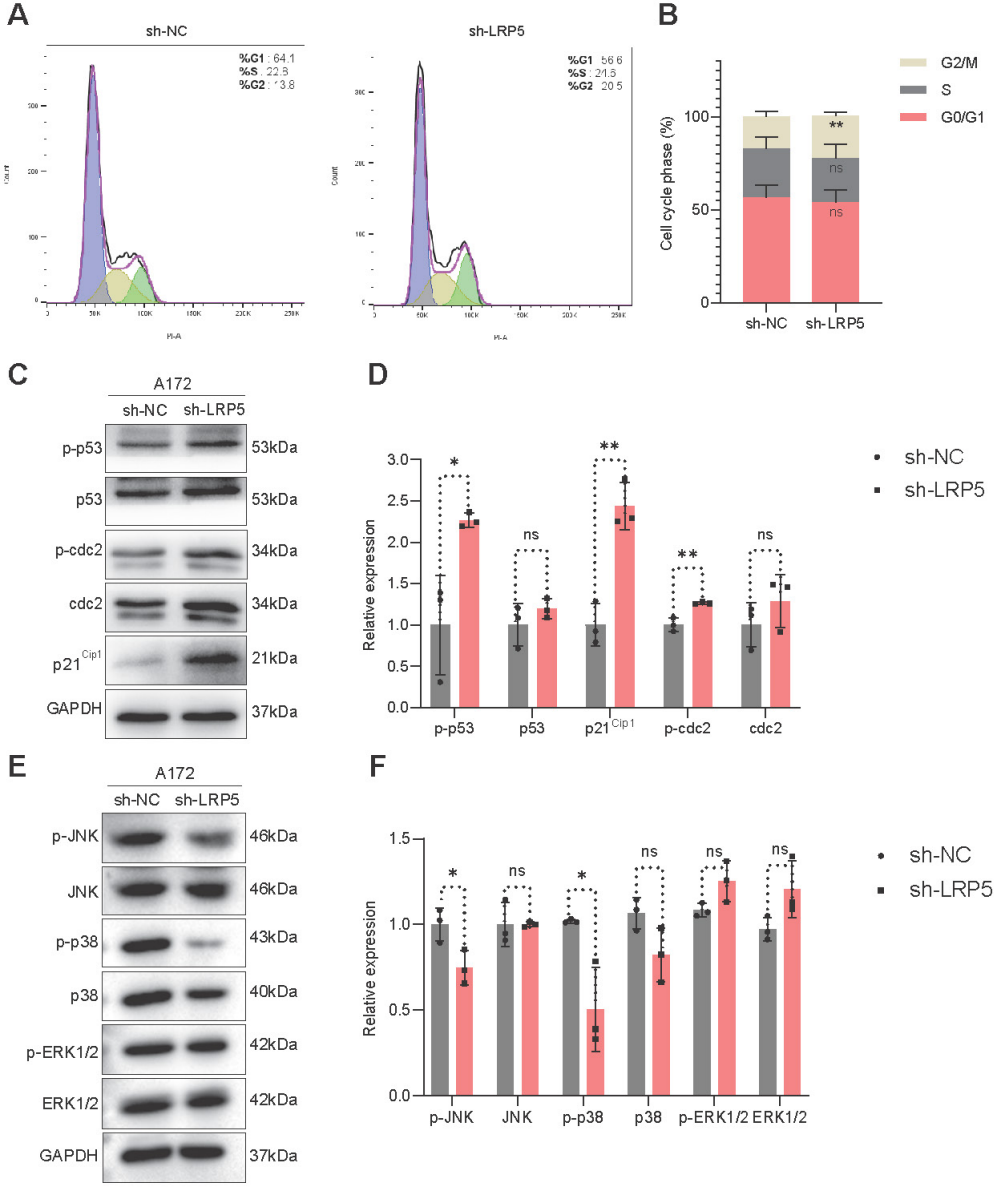
** LRP5 silencing induced glioma cells G2/M-phase arrest through the modulation of MAPK/p53/cdc2 pathway.** (A, B). The results of flow cytometry analysis of cell cycle. **vs sh-NC; ** *P*<0.01. (C, D). The protein levels of proteins associated with the G2/M phase transition (p53, p-p53, p21^Cip1^, cdc2, and p-cdc2) were analyzed by Western blotting. (E, F) The protein level of JNK, p-JNK, p38, p-p38, ERK1/2, and p-ERK1/2 was analyzed by Western blotting. ns P
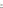
0.05, * P<0.05, ** P<0.01. All data were presented as the means ± SD.

To investigate the possible effect of LRP5 depletion on cell cycle progression, we utilized PI staining and flow cytometry to assess the distribution of cells in different phases of the cell cycle. We observed that the sh-LRP5 A127 cells group has a higher percentage of cells in the G2 phase (Figure 4A-B). This observation suggests that silencing LRP5 may lead to a decrease in cell proliferation by causing cells to be arrested in the G2 phase. We performed Western blotting to analyze the expression levels of cellular proteins associated with the G2/M phase transition. The outcomes revealed that when LRP5 was silenced, there was an upregulation of p-p53, p21^Cip1^, and p-cdc2 expression compared to the control group (Figure 4C-D). Overall, the above results established a connection between LRP5 and its influence on the p53/p21^Cip1^/cdc2 pathway, suggesting that LRP5 plays an important role in regulating the cell cycle by modulating the expression of key proteins involved in the transition from G2 to M phase. To explore the relationship between mitosis, mitogenic signaling pathways, and the LRP5 downregulation, we then assessed the phosphorylation levels of JNK, p38, and ERK1/2. The results showed that the levels of phospho-JNK(p-JNK) and phospho-p38(p-p38) were significantly downregulated in sh-LRP5 transfected A172 cells. However, the phosphorylation level of ERK did not exhibit any significant change (Figure 4E-F). These data suggest that LRP5 downregulation may induce cell cycle arrest at the G2/M phase via mitotic and mitogenic signaling.

### DKK-1 treatment inhibits the proliferation of glioma cells and the activation of the JNK and p38 pathways

**Figure 5:**
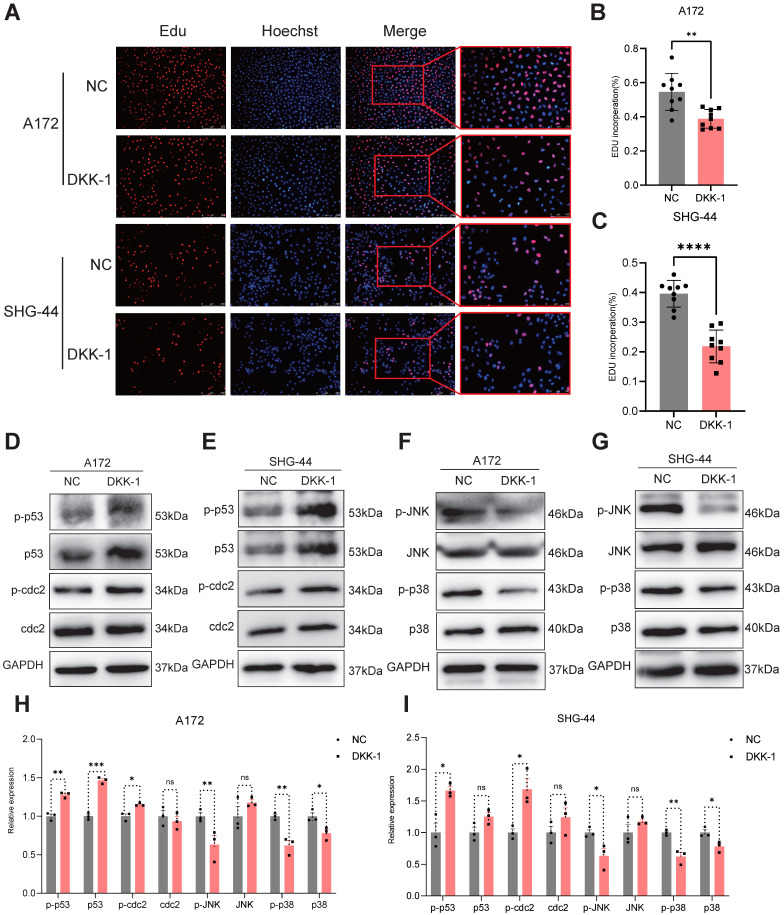
**The LRP5 antagonist DKK-1 reduced the proliferation of glioma cells through the modulation of MAPK/p53/cdc2 pathway.** (A-C) Representative images and the statistical results of Edu assay in A172 and SHG-44 cells (n=3). The nucleus was stained with Hoechst (blue), and the proliferating cells were stained with Edu (red). (scale bar=250μm) **P<0.01, ***P<0.001. (D-G) Representative images of protein levels for p53, p-p53, cdc2, p-cdc2, JNK, p-JNK, p38, and p-p38 were analyzed by Western blotting. (H, I) The statistical results of Western blotting in A172 and SHG-44 cells treated with DKK-1(0.5μg/mL) or a negative control for 48h. ns *P*
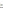
0.05, * *P*<0.05, ** *P*<0.01, ***P
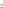
0.001. All data were presented as the means ± SD.

To investigate the effects of antagonists on glioma cells, we treated glioma cells (A172 and SHG-44) with or without the LRP5 antagonist DKK-1 (0.5μg/mL) for 48h. EdU assays revealed a decreased proportion of EdU-positive cells after DKK-1 treatment, indicating that cell proliferation was reduced (Figure 5A-C). Western blotting of proteins associated with the G2/M phase showed increased expression of p-p53 and p-cdc2 in the DKK-1 group (Figure 5D-E, H-I). These results suggested that DKK-1 induces G2/M phase abnormalities and inhibits cell proliferation. The results of the MAPK family-related proteins levels showed that the activation of p-JNK and p-p38 was reduced (Figure 5F-G, H-I). This shows that the effect achieved by the antagonist treatment is consistent with LRP5 downregulation.

## Discussion

Glioma, as a malignant brain tumor, becomes an extremely difficult condition to treat due to the particularity of its tumor environment.^18^, ^19^ The high recurrence rate among patients makes it necessary to explore and develop more drugs to provide additional treatment options. One of the main reasons for the poor treatment and high recurrent rate of glioma is the uncontrolled proliferation of cells within the tumor.^20^ Exploring the molecular mechanisms behind glioma is of great significance for drug development. In this study, we revealed that LRP5 is significantly upregulated in the brain tissue of patients with glioma. Suppression of LRP5 expression was observed to hinder the proliferation of glioma cells and consequently reduce the size of the tumor. Furthermore, our findings indicate that LRP5 influences the proliferation of glioma cells specifically during the transition from the G2 to M phase.

The classic Wnt signaling pathway is transmitted through the Frizzled (FZD) family receptors and LRP5/LRP6 co-receptors to activate the β-catenin signaling cascade. This pathway has the ability to modulate the cell cycle at various stages, thereby influencing cellular proliferation.^21^, ^22^ Currently, the primary medications utilized in the treatment of glioma encompass TMZ, doxorubicin (DOX), vincristine, nimustine (ACNU), and others. However, these drugs are constrained by issues related to drug resistance and adverse toxicities.^23^ LRP5 is a type I transmembrane protein and, together with LRP6, acts as a co-receptor and key component in the Wnt signaling pathway to transmit signals.^24^ Tao Tian and colleagues have found that blocking the Wnt signal pathway can inhibit the proliferation and invasion of glioma cells. It may achieve prevention of glioma cell metastasis in the clinic.^25^ A study has recognized LDLR as a potential target for the advancement of nanomedicines specifically designed for the treatment of glioma.^13^ Nanomedicines have nanoscale targeted delivery capabilities, which can enhance the bioavailability of drugs and reduce toxic side effects.^26^ They have been researched and developed for a variety of diseases.^27^-^30^ These advantages enable it to break through the blood-brain barrier and become a targeted drug with great potential for the treatment of brain tumors.^31^ However, there is a lack of research demonstrating the potential impact of LRP5 on the progression of glioma. In this research, we revealed a significant increase in LRP5 mRNA expression among glioma patients, and LRP5 exhibits a high discriminatory capacity in distinguishing between glioma and normal tissues. IHC confirmed a notably higher expression level of LRP5 in cancerous tissues compared to paracancerous tissues.

This study further found a positive correlation between tumor cell division activity, LRP5 expression levels, and the reduced recurrence-free time of patients, suggesting a potential association between glioma onset and progression and the upregulation of LRP5 expression. Therefore, the occurrence and progression of glioma are associated with the upregulation of LRP5 expression. DKK-1 is a typical antagonist of LRP5. Previous studies have found that DKK-1 can inhibit tumor growth 32, and our study also found that the proliferation of glioma cells in the DKK-1 treatment group was inhibited, along with changes in the expression of G2/M checkpoint proteins. This suggests that DKK-1 treatment can elicit the same cellular effects as those achieved through LRP5 knockdown, indicating that DKK-1 could be a potential therapeutic approach for targeting LRP5. Based on this, we believe that LRP5 may serve as a potential biomarker and therapeutic target for glioma, providing a scientific basis for the subsequent development of LDLR-targeted nanomedicines.

A study indicates that LRP5 can regulate cell proliferation in a mode independent of β-catenin and may serve as a regulatory factor in the YAP/TEAD signaling pathway in liver cancer.^33^ Xiaobo Nie and colleagues found that LRP5 promoted the development of colorectal cancer via canonical Wnt/β-catenin and IL-6/STAT3 signal pathways.^11^ Previous studies have not yet fully elucidated the effects of LRP5 on cell proliferation mechanisms in gliomas. The result of Spearman correlation analysis showed that the expression of LRP5 was positively correlated with cell proliferation ability. The sh-RNA lentiviruses were applied to down-regulated the expression of LRP5 in glioma cells (A172 and SHG-44 cells) to explore the influence of LRP5 on glioma cell proliferation. The findings indicated that silencing LRP5 led to decreased viability and slowed proliferation of glioma cells, indirectly underscoring the intimate relationship between abnormal glioma cell proliferation and increased LRP5 expression. The primary cause of abnormal tumor cell proliferation is dysregulation of cell cycle control.^34^ The impact of reduced LRP5 expression on glioma cell apoptosis was examined, with no statistically significant differences observed (data not included in this report for brevity). The results of flow cytometry revealed that, following LRP5 downregulation, a greater proportion of cells were arrested in the G2 phase. This suggests that LRP5 may promote glioma cell proliferation by regulating the G2 to M phase transition.

The cell cycle checkpoint is crucial for cell division and proliferation.^35^ The Cyclin B-cdc2 complex, also known as cyclin-dependent kinase 1 (CDK1), plays a vital role in the transition from the G2 phase to the M phase of cell division.^36^ The phosphorylation of cdc2 leads to its inactivation, preventing cells from transitioning smoothly and resulting in their arrest in the G2 phase.^37^ Our results demonstrate an increase in the levels of p-p53, p21^Cip1^, and p-cdc2. Therefore, the Cyclin B-cdc2 complex was inactivated, causing cells to remain arrested in the G2 phase. This finding further indicates that knocking down LRP5 induces a cell cycle blockade in glioma cells at the G2/M phase, ultimately leading to a reduction in glioma cell proliferation. Studies have found that changes in LRP5 expression can lead to alterations in the activity of the MAPK signaling pathway^10^, ^38^. The MAPK family regulates various cellular activities, and it consists of four major subfamilies: ERK, JNK, p38, and ERK5.^39^ The JNK and p38 pathways are closely related to cellular stress response, inflammation, and apoptosis. Notably, the activation of p38 is reduced in various cancer cells but is elevated in glioma cells.^40^ Abnormal activation of ERK1/2 is observed in various cancers; however, it can also induce apoptosis under specific conditions.^41^ Studies have shown that the activation of the ERK pathway can promote glioma cell proliferation and is associated with resistance to EGFR inhibitors in glioma cells.^42^ In our study, the phosphorylations of JNK and p38 in glioma cells were reduced with LRP5 downregulation or DKK-1 treatment, suggesting that LRP5 may affect the activation of these two pathways. Therefore, we suggest that MAPK/p53/cdc2 may be a potential mechanism through which LRP5 regulates the cell cycle and affects the proliferation of glioma cells. Whether the reduction in cell proliferation and the cell cycle arrest induced by silencing LRP5 is dependent on the inhibition of the JNK and p38 MAPK pathways remains to be investigated in future studies.

## Conclusions

In summary, our study suggests a notable upregulation of LRP5 expression in glioma tissue. Both knocking down LRP5 and using antagonists can affect the proliferation of glioma cells from the G2 phase to the M phase and inhibit MAPK/p53/cdc2 pathway. These findings offer novel perspectives for the design of pharmaceutical agents aimed at modulating LRP5 expression as a therapeutic approach for treating glioma.

### Author contributions

Ying-Yi Feng: Performed the experiments, analyzed the data, and wrote the paper.

Xin Jin: Performed the experiments and provided paraffin sections of brain tissue.

Min-Xuan Pan; Jia-Min Liao: Analyzed and interpreted the data.

Xian-Zhang Huang; Chun-Min Kang: Conceived and designed the study.

All authors had read and approved the final manuscript.

### Funding

This work was supported by the State Key Laboratory of Dampness Syndrome of Chinese Medicine (No.SZ2022KF18), the Project of Administration of Traditional Chinese Medicine of Guangdong Province of China (No.20222081), Special Project of Dampness Syndrome of Traditional Chinese Medicine Bureau of Guangdong Province (No.20225005), the Natural Science Fund of Guangdong (No. 2021A1515111125), Key Project of State Key Laboratory of Dampness Syndrome of Chinese Medicine (No.SZ2021ZZ32), Science and Technology Planning Project of Guangdong Province (No.2023B1212060063), and the Foundation of Medical Science and Technology Research of Guangdong Province (No.A2022539, No.2024050), Special Funding for Scientific and Technological Research on Chinese Medicine at Guangdong Hospital of Traditional Chinese Medicine (YN2024GZRPY047).

### Data availability statement

The data used and analyzed in this study are available from the corresponding author upon reasonable request.

### Ethics declaration

This study was reviewed and approved by the Ethics Committee of Guangdong Sanjiu Brain Hospital. The approval number is 2021-01-084 and the approval data is 2021-11-24. Written informed consent was obtained from all participants.
